# Significance of VLPs in Vlp-circRNA vaccines: a vaccine candidate or delivery vehicle?

**DOI:** 10.1080/15476286.2024.2399307

**Published:** 2024-09-06

**Authors:** Reeshu Gupta, Kajal Arora, Nupur Mehrotra Arora, Prabuddha Kundu

**Affiliations:** a Department of Research and Development, Premas Biotech Pvt Ltd., Industrial Model Township (IMT), Gurugram, India; b Research and Development Cell, Parul University, Vadodara, Gujarat, India

**Keywords:** Circular-RNAs, VLP, vaccine, delivery of circRNAs, CircRNA vaccine

## Abstract

Circular RNAs (circRNAs) are a class of single-stranded RNAs with a closed loop lacking 5ʹ and 3ʹ ends. These circRNAs are translatable and, therefore, have a potential in developing vaccine. CircRNA vaccines have been shown to be more stable, safe, easy to manufacture and scale-up production when compared to mRNA vaccines. However, these vaccines also suffer from several drawbacks such as low circularization efficiency for longer RNA precursor and usage of lipid nano particles (LNPs) in their delivery. LNPs have been shown to require large amounts of RNA due to their indirect delivery from endosome to cytosol. Besides, individual components of LNPs provide reactogenicity. Usage of virus like particles (VLPs) can improve the increased production and targeted delivery of circRNA vaccines and show no reactogenicity. Moreover, VLPs has also been used to produce vaccines against several diseases such as hepatitis C virus (HCV) etc. In this article, we will discuss about the methods used to enhance synthesis or circularization efficiency of circRNA. Moreover, we will also discuss about the significance of VLPs as the delivery vehicle for circRNA and their possible usage as the dual vaccine.

## Introduction

RNA vaccine technology uses RNA injection, which generates antigenic proteins via using the body's own translation machinery; therefore, there has been significant progress in vaccine development in the past few years. However, it has several limitations, such as instability owing to the short half-life of mRNA, local reactogenicity, cost, cold storage requirements, and transportation facilities. These issues have become crucial in controlling pandemics and, therefore, lead to the synthesis of circRNAs-based vaccines. Recently, Coalition for Epidemic Preparedness Innovations (CEPI) funding has been provided to the Houston vaccinology team to advance the 'circRNA' platform against future epidemic and pandemic diseases (https://cepi.net/promising-new-circrna-vaccines-explored-cepi-hmri-collaboration).

CircRNAs vaccines belong to a single-stranded RNA having closed-loop due to the absence of 5' cap and 3' poly A tail. On the basis of exonic and intronic sequences, circRNAs are typically classified into four main types: (1) Exons containing exonic circRNAs. Circularization occurs because the complementary regions of introns facilitate back-splicing. (2) Exonic-intronic circRNAs that retain introns along with exons in circRNAs and (3) intronic circRNAs that contain only introns. These are formed owing to the presence of AU regions at the 5' splice site and the C-rich domain near the breakpoint. The GU-rich domain protects the C-rich domain from degradation, thus generating intron-derived circRNAs and (4) tRNA intronic circRNAs that are generated by the cleavage of pre-tRNAs by the tRNA splicing endonuclease (TSEN) complex [1]. In comparison to linear mRNA vaccines, the absence of 5' cap and 3' tail region protects circRNAs from degradation by RNases and thus do not require the cold chain equipment that are the major limitations of mRNA vaccines in pandemics. The circRNA vaccines offer greater stability compared to linear mRNA vaccines. The stability of mRNA vaccines can be enhanced by nucleotide modification of the mRNA backbone and untranslated regions. However, these modifications complicate manufacturing processes and thus enhance the cost of goods, in addition to requiring cold chain storage equipment [2]. CircRNA vaccines prolong antigen presentation due to the antigen retention by antigen-presenting cells, thus triggering both cell-mediated and humoral immune responses more effectively. Moreover, N6-methyladenosine (m6A) RNA modification of human circRNAs inhibits innate immunity and results in low immunogenicity. Inhibition of innate immunity by circRNAs is essential because mammalian cells possess innate immunity to detect foreign circRNAs due to the presence of pattern recognition receptors (PRRs) such as retinoic acid-inducible gene-I-like receptors (RLRs). The activation of RIG-I by circRNAs induces the cytokine storm and severe inflammation [3]. It has been shown that circRNAs with exogenous sequences are capable of inducing cytokine storms [4]. For instance, it was shown that approximately 80 circRNAs were either up‐regulated or down‐regulated in the case of liposaccharide-induced sepsis. These circRNAs containing exogenous sequences induce several pathways that may contribute to a cytokine storm, such as TNF or NF-kB pathways [5]. CircRNAs having endogenous sequences do not induce cytokine storm. Due to their low immunogenicity, purified artificial circRNAs are considered suitable for vaccine development. Notably, the synthesis of circRNAs using endogenous sequences produces a small amount of protein and low circularization efficiency for longer precursor RNAs [2]. Therefore, methods should be developed to produce large amounts of proteins using endogenous sequences to evade innate immunity.

**Figure 1.**
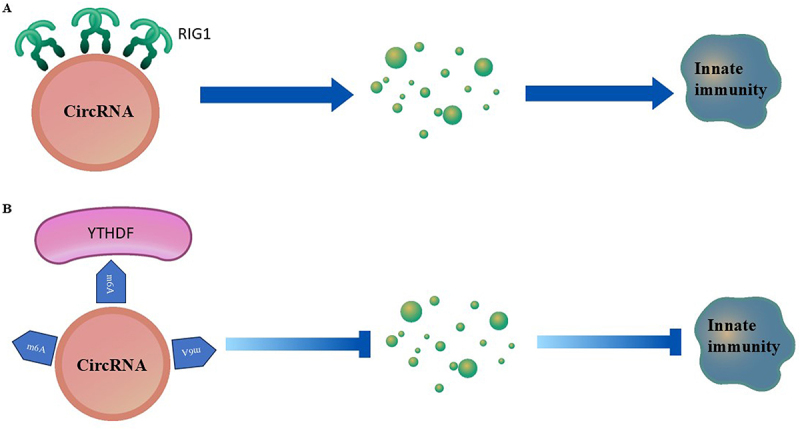
Innate immunity with unmodified and modified circRNAs a) unmodified circRNA binds to RIG1 and release cytokines that induce innate immunity b) m6A modification of the circRNA recruit YTHDF which prevents binding of circRNAs to RIG1.

**Figure 2.**
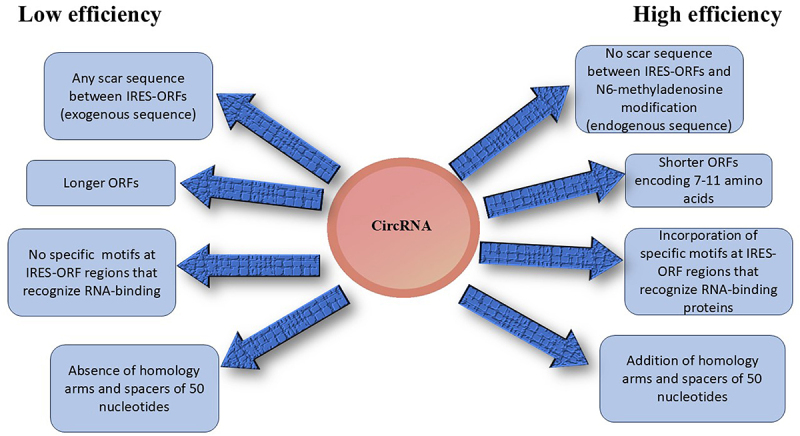
Comparison of the attributes of low- and high-efficiency circRnas.

The synthesis of circRNAs requires the design of linear RNA precursors generated by an in vitro transcription (IVT) method. T7 or SP6 RNA polymerases were used in the IVT. For the synthesis of m6A modified circRNAs, amplification buffer having N6-methyl-ATP (m6ATP) should be added. The core element of the linear RNA precursors is the IRES-ORF cassette. IRES are internal ribosome entry sites with special sequences located upstream of the start codon of the open reading frame (ORF). These sequences engage 40 S-ribosomal units to induce translation in a cap-independent manner. ORF elements are located downstream of IRES and encode customized proteins or peptides. These elements are crucial for storing coding information because they have both start and stop codons. Protein synthesis begins and terminate by recognizing the start codon of the 40 S ribosomal subunit and stop codons of the ORF, respectively [2]. This process was repeated until all the circRNAs were degraded. Other elements that facilitate circularization and reduce innate immunity also play important roles. Any scar sequence between IRES-ORFs reportedly reduces the translation efficiency. Longer circRNAs are prone to nicking and thus reducing the length of IRES, and ORF will enhance translation efficiency. For instance, Kozak and AU-rich sequences may eliminate this issue [6]. In eukaryotes, the RNAs include the Kozak consensus sequence, GCCAUGG, which contains an initiation codon and conserved flanking nucleotides. The Kozak sequence plays a major role in initiation of translation in eukaryotic systems. Any mutation in the Kozak sequence of circRNAs can reduce the efficiency of translation initiation. For instance, the weaker Kozak sequence within the FLAG-EGF circRNAs produced a translation product with less intensity than the strong Kozak sequence. The sequence of FLAG peptide is DYLDDDDL and is encoded by 24 nucleotides [6]. Typically, IRES are 1000 nucleotides longer. Strategies, such as ribosome profiling (library construction), are the best tools for identifying shorter ORFs. Shorter ORFs encoding 7–11 amino acids may be beneficial in inducing desirable immune responses. Further incorporation of specific motifs at IRES-ORF regions that recognize RNA-binding proteins can enhance translation efficiency. For example, poly (A)-binding proteins induce the binding of eukaryotic initiation factors (eIFs), poly(C)-binding protein motifs induce the recruitment of ribosomal proteins, and m6A motifs induce YTHDF and initiation factors required for protein translation. It was shown that m6A modification of circRNAs recruit YTHDF2, which prevents the activation of RIG1 and therefore helps the host differentiate self from foreign circRNAs (Figure 1). Moreover, the addition of homology arms and spacers of 50 nucleotides promotes the circularization of circRNAs [3]. A comparison of the attributes of the low- and high-efficiency circRNAs is shown in Figure 2.

**Figure 3.**
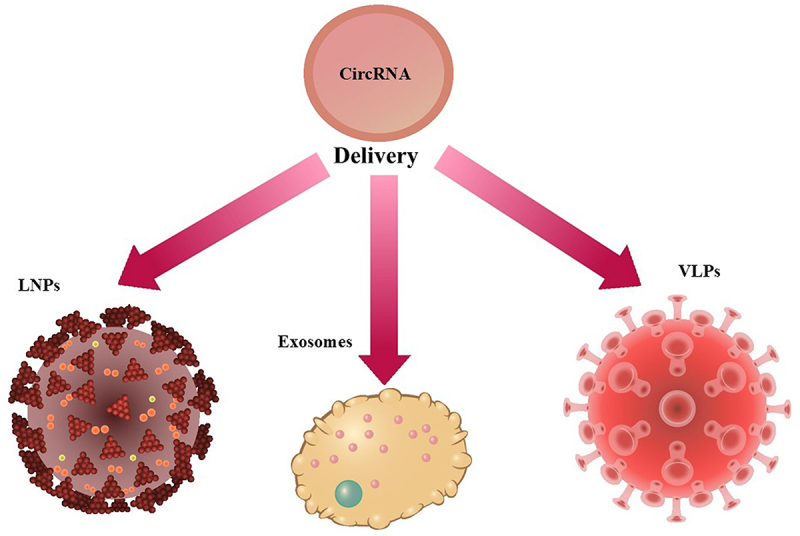
Delivery of circRNAs by a) LNPs and b) exosomes c) VLPs.

Several techniques have been used to synthesize and deliver circRNAs (Figure 3). Lipid nanoparticles are primarily used for the delivery of circRNAs, which showed reactogenicity. Intramuscular and intravenous injections of LNPs showed accumulation in the liver, causing liver injury [7]. Therefore, there is a need to establish methodologies to enhance the translation efficiency of circRNAs and to develop suitable delivery vehicles. Recently, VLPs-circRNAs have been shown to enhance prolonged antigen presentation and, thus, increased immune responses [8]. In view of this, in this article, we will discuss the various methodologies used to synthesize and deliver circRNAs, with an emphasis on the usage of VLPs in circRNA-based vaccines.

## Synthesis of CircRNAs

Circularization can be performed using various methods after the synthesis of a linear mRNA precursor. For example, circRNAs are synthesized by ligating the 3’0 H splice acceptor and 5'-phosphate splice donor ends using chemical, enzymatic, or permuted intron exon (PIE) methods. In chemical ligation methods, chemicals such as cyanogen bromide (BrCN) are used to ligate the 3' and 5' ends [9,10], whereas in the case of the enzymatic ligation method, RNA ligases are used [11]. RNA ligase I is used to circularize linear RNA precursors containing less than 500 nucleotides with no secondary structure. The ligation efficiency of RNA ligase II is comparatively higher than RNA ligase I because it first transfers adenosine to 5'-phosphate [12], which is then attacked by the 3’0 H group and thus form phosphodiester bond. Both chemical and enzymatic reactions use complementary templates that bridge the 3’0 H splice acceptor and 5'-phosphate splice donor sites in the linear mRNA precursor. Subsequently, DNase degrades template DNA. T4 RNA ligase is used to circularize the RNA using splints, but the yield is lower because of intermolecular polymerization. Splints are complementary sequences to both the 3'0 H and 5'P groups of the mRNAs. Subsequently, these splints are removed using DNase. Recently, it was shown that T4 RNA ligase can be used to convert any RNA into rings without significant limitations in size and sequence. In this method, RNA is hypothetically cut at a new site near the ligation site, so that few base pairs are formed near the ligation site. This RNA was used by T4 RNA ligase to generate circRNAs. In this method, splints were not required and the yield of circRNAs was high. Dilution of T4 RNA ligase buffer can further reduce the formation of by-products [12]. The main advantage of chemical and enzymatic ligation methods is the introduction of endogenous sequences, which helps in inhibiting innate immunity. However, the low circularization efficiency of longer precursor RNA, is a major disadvantage of both the methods. Other drawbacks include low throughput, unexpected products, and complicated procedures are other drawbacks [2]. Unexpected products can be generated by the reversal of RNA ligases. This could be due to the strong preference for hydrolysis of the 3'-terminal phosphodiester bonds, generating 3'-phosphate [11].

The most common approach used for circularization of mRNA is the usage of ribozymes and is achieved through PIE system that mediate consecutive ester exchange reactions. In case of group I intron self-splicing, a series of ester exchange reactions are required [13]. During splicing, 3’0 H of exogenous GTP attacks the 5' splice site and form exon 1 (E1) with a free 3’0 H group along with the excision of 5' intron. Subsequently, the free 3’0 H group of E1 attack the 3' splice site of exon 2 (E2), resulting in ligation of E2 and E1 with the excision of 3' intron. However, it has a limitation of contaminated 80 nucleotides of E1 and E2 sequences at the end joining of circRNAs. Compared to the group I intron PIE system, group II introns circularized linear RNAs without E1 and E2 scar sequences. In the case of group II introns, 2'0 H of internal adenosine of intron form lariat structure that contain 2'5' and 3'5; phosphodiester bonds. Afterwards, 3'0 H group of exon attack the 3' splice site and ligate exons with the release of introns as a lariat [14]. The major disadvantages of the PIE system are the introduction of exogenous sequences and the presence of contaminants after synthesis. Circular mRNA generated by the back-splicing system utilizes complementary intronic sequences that facilitate intron–intron interactions that subsequently lead to back-splicing events. However, the main product of back splicing could be the linear splicing by-product, where proteins are formed from these by-products even in the absence of circRNAs [8,15].

In another method endogenous RNA ligase (RtcB) is used and this method is described as ribozyme assisted circRNAs (racRNA) method. In this method, linear RNA precursor contains an RNA of interest flanked by ribozymes that undergo spontaneous autocatalytic cleavage and generate 5' and 3' ends that are then ligated by RtcB. Most ribozymes require several minutes or even hours for the complete cleavage. This is not useful because half-life of RNA is short. Therefore, RNA will degrade before the generation of 5' and 3' ends. In racRNA method, Twister” ribozymes are used because they generate 5' 0 H and 2',3'-cyclic phosphate ends at much higher rate than other ribozymes. Following this, the RNA between the ribozymes circularizes and form circRNA. This system is also known as Tornado system (Twister-optimized RNA for durable overexpression) and has been used to produce high number of circular mRNAs in mammalian cells when compared to mRNA produced by the backsplicing system. In case of using Pol III promoter, any uracil-rich termination sequences in mRNAs should be avoided to warrant transcription of the full-length RNA. These racRNA contain binding sites for microRNA and can elicit cytotoxicity [16]. Therefore, further studies are required to check their toxicity *in- vivo*. Moreover, finding the best IRES and promoters using different combinations can further enhance the high protein output in mammalian cells. For example, five elements (vector backbone, 5' and 3' untranslated regions, IRES and synthetic aptamers recruiting translation initiation machinery) has been shown to enhance translation for more duration over IVT, across multiple transgenes [17]. Gene lacking the stop codon will generate continuously translated circular mRNA (CTC mRNA) which have shown to enhance production of multimeric polyproteins in bacterial cell-free expression systems. However, there are several issues if homogenous post-translationally modified proteins are required. Incorporation of 2 A ‘self-cleavage’ peptide motif can overcome this problem by co-translational cleavage of nascent polypeptide chains [18]. 2A self-cleavage motifs were first discovered in a foot and mouth disease virus. 2A refers to a specific peptide sequence that induces ribosome skipping during translation. Ribosomes fail to form peptide bonds at these sites and resume translation. This process is known as ribosome skipping, and it helps in the generation of functional proteins from a single ORF. The members of the 2A family were named after the virus has been described. The typical sequence of 2A is DxExNPGP. Cleavage of ‘2A’ motifs is universal in eukaryotes; however, the efficiency of 2A motifs can differ. Rolling circle translation of recombinant human erythropoietin produced large multimeric variants, and incorporation of a 2A self-cleavage motif resulted in the production of multimeric proteins due to ribosome skipping at the 2A site, resulting in the highest levels of secreted proteins [18].

## Delivery of circRNAs

### LNPs and associated challenges

One major challenge in case of RNA vaccines is the targeted delivery to the specific cell types. After the synthesis of mRNA using IVT methods, it is packaged into liposomes or lipid nanoparticles. After administration, these mRNAs entered in the liver cells. Typically, absence of positive charge in the reticuloendothelial system in the liver allows the maximal delivery of mRNA vaccines to hepatocytes via ApoE-dependent, receptor-mediated endocytosis [19]. It has been reported that ionized LNP are capable of delivering nucleic acids to hepatocytes by binding with Apolipoprotein E (ApoE). ApoE binds to low-density lipoprotein receptors (LDLR) and delivers nucleic acids to the liver. LDLR belongs to evolutionarily conserved low-density lipoprotein (LDL) receptor family and are highly expressed by hepatocytes [19,20]. This is an active liver-targeting delivery mechanism. Moreover, this is a matter of concern where mRNA therapeutics require delivery of mRNA to other tissues than liver. It has also been shown that accumulation of mRNA in liver may lead to liver injury [7]. Another disadvantage is reactogenicity of LNPs. Therefore, it is wise to develop methods that can deliver mRNA at the desired site and decrease the reactogenicity of LNPs.

The key components of lipid nanoparticles are ionizable lipids, helper lipids, cholesterol and polyethylene glycol (PEG)-lipids. These components are capable of inducing several types of toxicity and reactogenicity. Cationic ionizable LNPs and cationic ionizable lipids due to their positive charge interact with the anionic RNA during particle formation and exhibit neutral charge at pH 7.4. The examples of cationic ionizable LNPs are neutral phospholipids, cholesterol, PEG-lipids and examples of cationic ionizable lipids are (1,2-dioleoyloxy-3-trimethylammonium propane chloride (DOTAP), 1,2-dio-leoyl-sn-glycerol-3-phosphoethanolamine (DOPE) and *N*-[1-(2,3-dioleoyloxy) propyl]-N, N, N-trimethylammonium chloride (DOTMA). Afterwards, due to the low pH at the time of internalization, these lipids acquire positive charge and facilitate membrane fusion and leads to the formation of endosomes. Similarly, PEG-lipid provides hydrophilic environment at the surrounding of LNPs which enhance LNP stability by sterically hindering the aggregation of LNPs during storage. Cholesterol has also been shown to improve stability and delivery of mRNA vaccines. The examples of neutral phospholipids are 1,2-distearoyl-sn-glycero-3 phosphocholine (DSPC) which provide bilayer structural stability [21]. LNPs increased the encapsulation efficiency of RNA and protect it from degradation by endosomal enzymes. LNP fuses with the target cell membrane and form the endosome in the cytoplasm via TLR4-mediated endocytosis. Afterwards, negatively charged lipids in endosomes interact with the positively charged lipids of LNPs and loosen the endosomal membrane by forming non-bilayer structure and releases the RNA from endosome. Modification of the hydrophilic head and tail ends of cationic lipids can further enhance the endosome escape or vaccine delivery such as in the case of YSK12-C4, pH-sensitive cationic lipid [22]. An adequate combination of a hydrophilic head and hydrophobic tail can be used to select appropriate lipid particles that can facilitate endosomal escape and enhance the availability of siRNA to target cells. For example, LNP composed of a pH-sensitive cationic lipid CL4H6 (CL4H6-LNPs) showed efficient gene silencing activity, endosomal escape, cytosolic release, and high biodegradability [22]. Delivery can be further improved by charge-altering releasable transporters (CARTs) which are temporarily cationic molecules and have been applied for the consistent delivery of circRNA over 96 h [17,23]. It was shown that circRNA delivered with CARTs can acquire the normal range (2.8 mIU/ml to 17.9 mIU/ml) of erythropoietin in 4 days in mice. However, CARTs have not been optimized for circRNA transport; therefore, further optimization is required [17]. Moreover, biodegradable lipidoids with eight ester bonds also called AX4-based lipid nanoparticles (AX4-LNPs) rapidly degrade in spleen cells and thus, expedite the expel rate of circRNA vaccines from endosomes [24]. Direct delivery to the lymph node or dendritic cells can prevent hepatic damage including hepatitis and control side effects [2].

Although LNPs are made up of several kinds of lipids, it has been shown that ionizable lipids and PEG-lipids show highest reactogenicity. Ionizable lipids such as SM-102 and ALC-0315 have been used to package Moderna and Pfizer-BioNTech COVID-19 vaccine, respectively [25]. These vaccines showed several mild to moderate side effects including fever, headaches, fatigue etc. The side effects are more common following the second dose of the SARS-CoV-2 vaccine. The cationic charge of these lipids interacts with the negative charge of the endosome membrane, which may damage the endosomal membrane and induce non-wanted immune cells activation. Similarly, PEG-lipids evade the immune response and prevent opsonization by complement molecules and thus lead to the production of anti-PEG antibodies [26]. The production of anti-PEG antibodies is more common in the case of PEG having large molecular weight. Therefore, it can be inferred that individual components of LNPs can trigger reactogenicity and immune responses. However, it is difficult to analyse the consequences generated by the individual components of LNP carriers (eLNPs) because the assembled eLNPs play major role in delivering nucleic acids and not the individual components. Another disadvantage is the usage of eLNP carriers as a control in mRNA-LNP vaccine. This is because they differ from mRNA-loaded LNPs in terms of size, composition, fusion, altered concentrations of helper lipids, interference from ionizable lipids, positive surface charges, splitting dynamics, and surface interactions. Therefore, researchers need to use placebo, PBS, naked nucleic acids etc, as the other controls [27]. Several factors influence the loading of mRNA to LNPs, such as the molar ratio of PEG-lipids, lipid-to-mRNA mass ratio, and N/P ratio (positively charged nitrogen of ionizable lipids and negatively charged phosphates of nucleic acids) [28]. The binding of the eLNPs and mRNA-loaded eLNPs differed due to their differential interaction with homing proteins such as ApoE. Therefore, it may also initiate local and systemic reactogenicity [28]. Food and Drug Administration (FDA) has approved LNPs having similar structural components to deliver vaccines. Therefore, evaluation of reactogenicity is essential to expand the clinical scope of LNPs. The reactogenicity can be assessed by behaviour observations, haematological studies, histopathology or by measuring inflammatory markers. However, LNPs have several other limitations, such as low biocompatibility, complex production methods, and difficulties in the production at larger scale. Considering these points, it could be predicted that usage of LNPs in circRNA vaccines may show effective results at preclinical levels but they may fail to translate as a vaccine at the clinical levels.

Some examples of vaccines having LNPs as a carrier along with their composition and side effects are given in Table 1.

**Table 1.** LNPs composition and side effects in various vaccines.

SN	LNPs	Vaccines	Disease	Side effects
1.	Lipids, 1,2-distearoyl-sn-glycero-3-phosphocholine (DSPC), cholesterol, and 1,2-dimyristoyl-rac-glycerol, methoxypolyethylene glycol (PEG2000-DMG) [29]	Modified mRNA encoding the protein for human erythropoietin (hEPO)	Anemia	Increased white blood cell counts, changes in the coagulation parameters at all doses, as well as liver injury and release of interferon γ–inducible protein 10 in high-dose groups
2.	Ionizable amino lipid, phospholipid, cholesterol and a PEGylated lipid [30]	Non-modified mRNA encoding rabies virus glycoprotein G (RABV-G),	Rabies	Injection site erythema and oedema, cytokine generation
3.	Acuitas ALC-0315 combined with DSPC, cholesterol and a PEG – lipid [31]	Pfizer/BioNTech vaccine	COVID-19	Anaphylaxis
4.	Lipid H (SM-102) [32,33]	mRNA-1273 moderna	COVID-19	Anaphylaxis
5.	DLin-MC3-DMA, Distearoylphosphatidylcholine, cholesterol, and the PEGylated lipid DMG-PEG 2000 [34,35]	Onpattro	Polyneuropathy caused by an illness called hereditary transthyretin-mediated amyloidosis	infusion-`related reactions
6.	Phosphatidylcholine (Avanti Polar Lipids, Alabaster, AL), cholesterol (Calbiochem, San Diego, CA), and N-methoxy-PEG (molecular weight, 1,900)-oxycarbonyl-distearoylphosphatidylethanolamine (DSPE; Genzyme, Cambridge, MA) [36]	MM-302	Breast cancer	Cardiac toxicity

### Naked delivery

CircRNAs can also be delivered as the naked circRNA without requiring any delivery system. In this strategy naked circRNAs are dissolved in Ringer's solution. Ringer's solution contain calcium ions, which facilitates RNA uptake by antigen presenting cells such as dendritic cells [37]. Hence, intradermal administration can be considered the feasible route for the delivery of circRNA due to the presence of antigen presenting cells (APCs) in the skin. Percutaneous administration using ultrasound-guided percutaneous injection deliver vaccine to lymph nodes and trigger T-cell responses [38]. The literature demonstrated the use of gene guns, electroporation, and microneedles to improve the efficacy of naked mRNA antigen presentation [39]. The same methods can be used to deliver the circRNAs.

It has been shown that circular mRNA, termed cmRNA containing internal ribosome entry site element and newly designed homology arms and RNA spacers, is suitable for direct intratumourally administration of cmRNA to treat cancer. This cmRNA encodes a mixture of cytokines and enhanced anti-programmed cell death protein 1 (PD-1) antibody-induced tumour repression [40]. However, these approaches are at the preliminary stages and not much data are available about naked delivery of circRNA vaccines.

### Exosomes mediated delivery

Exosomes are extracellular vesicles having the size of nanometre in range thus can serve as nanocarriers. These vesicles have natural affinity to recipient cells and can shuttle circRNA between cells. Thus, exosome mediated delivery can overcome several obstacles encountered in circRNA delivery. Exosome-mediated mRNA delivery for SARS-CoV-2 vaccination [41] and to inhibit drug resistance in glioma cells [42] has recently been reported. However, exosomes mediated delivery has several limitations. For example, exosomes can load only the small number of RNAs, and thus require large cell culturing followed by RNA isolation [43].

The presence of WW domain on extracellular vesicles allows viral membrane antigens to be selectively recruited onto the surface of WW domain – activated (WAEVs). The WW domain is a modular protein structure that recognizes the proline-rich Pro-Pro-x-Tyr (PPxY) motif in specific target proteins [44]. Budding of WW domain-containing extracellular vesicles requires secretory carrier-associated membrane protein 3, has a PPxY motif, and therefore interacts with WW domains to recruit fused influenza and HIV membrane antigens onto extracellular vesicles. Immunization with these extracellular vesicles induces immune responses [45]. WW domain-containing extracellular vesicles can be used to display membrane antigens of various types of enveloped viruses.

### VLPs as a delivery vehicle for circRNAs

VLPs compose of the major structural proteins of a virus that are needed to form viral capsid, but lacks viral genomic material. VLPs are used as the delivery vehicles of mRNA vaccines due to several advantages, such as targeted delivery, biocompatibility, cell penetration, and easy degradation via proteolytic mechanisms. The bacteriophage MS2 VLPs can self-assemble by forming interaction with the coat proteins of bacteriophage and 19 nucleotide MS2 cistron [46]. These RNAs can be delivered to the target cell. Similarly, vitamin folic acid (FA) can be covalently linked to the surface of cowpea mosaic virus (CPMV) and Hibiscus chlorotic ringspot virus (HCRSV) VLPs to achieve cell-specific delivery [47]. The structural proteins of different adenoviruses can generate VLPs of different sizes. For instance, adenovirus dodecahedron (Dd) VLPs can successfully deliver oncogene inhibitors to inhibit hepatic cancer and significantly decrease the expression of oncogenes [48]. The use of Dd requires the low effective dose and thus can reduce cytotoxicity. For instance, in comparison to the free bleomycin, lower dose of bleomycin is required while using adenovirus dodecahedron VLP. Bleomycin is an anticancer antibiotic [49]. In comparison to LNPs, VLPs have several advantages. The main benefit is that the surface proteins of VLPs can be replaced with the surface proteins of other virus making its application broad. Another advantage includes direct delivery of VLP to cytosol instead of endosomes and thus it prevents undesired immune activation due to the cellular damage of endosomal membrane. Because the endosomal route is not required, relatively small number of mRNAs is required to achieve mRNA expression [8]. Therefore, VLPs can also be used to deliver the circRNAs to specific cell types and can provide durable immune response.

While using VLPs as the delivery vehicles, mRNAs are first expressed in mammalian cells and then they are directed to enter VLPs during assembly. The VLP-mRNA vaccines are more advantageous when self-amplifying RNA in packaged in VLPs because they have been shown to enhance robust immune response [50,51]. The DNA template of the self-amplifying RNA vaccine contained conserved sequence elements, non-structural proteins 1, 2, 3, and 4, subgenomic promoters, and vaccine immunogens. Non-structural proteins are indispensable for the formation of RNA-dependent RNA polymerase (RdRP) complexes. Therefore, multiple copies of RNA are produced after in vitro transcription, leading to increased expression. Due to the high expression of the vaccine immunogen, self-amplifying RNA can be delivered at low doses in comparison to conventional vaccines. There are various ways by which the efficacy of self-amplifying RNA can be achieved, such as using a variety of RdRP or capsid proteins, incorporation of chemically modified nucleotides, sequence optimization, and different purification strategies etc [52]. For instance, it was shown that self-amplifying mRNA was protected in a viral protein capsid from the plant virus Cowpea Chlorotic Mottle Virus (CCMV), which can package a large variety of heterologous ssRNA with 2500-4200nt into wildtype capsids. They evaluated the efficacy of this platform for delivering the ovalbumin epitope SIINFEKL, which was coupled to RNA-dependent RNA polymerase from the Nodamura insect virus. Incubation of immature DCs with these VLPs results in increased activation of maturation markers, enhanced RNA uptake/replication levels, dendritic cell activation, and a significant increase in antigen-specific T cells [50]. Similarly, self-amplifying RNA was packaged using capsid proteins of spherical and cylindrical viruses. To make RNA self-amplifying, RNA was fused with the RNA dependent RNA polymerase of Nodamura virus [53]. The results suggest that both cylindrical and spherical VLPs can deliver mRNA genes to immune cells and significantly enhance their expression. Continuous translation occurs due to the absence of stop codon in the ORF. However, continuous RNA production was shown to have no effect on protein output while using tornado system. It may be because of presence of many stop codons in most IRES sequences [8]. A COVID-19 vaccine, VLPCOV-02, was developed, with several modifications to VLPCOV-01. VLPCOV-01 is an LNP-encapsulated saRNA platform containing non-structural proteins 1–4 of alphavirus and a membrane-anchored receptor-binding domain (RBD). These modifications include the incorporation of the pan HLA DR-binding epitope sequence, a short stretch of peptide that binds to the most common human leukocyte antigen – DR isotypes, and 5-methylcytosine. Incorporation of these modifications into the vaccine lowers reactogenicity and adverse events in phase I clinical trials, along with robust induction of antibody responses [28].

In few studies, env and gag proteins of HIV was used to form gag based VLPs delivering mRNA. For example, the native cytoplasmic tail of the SARS-CoV-2 spike protein was fused with the CT of the gp41 transmembrane env protein of either human immunodeficiency virus (HIV-1) or simian-human immunodeficiency virus (SIV), producing a hybrid Spike protein. The truncated form of retroviral CT at 745 amino acid induced cell surface expression of the spike protein. Co-transfection of mRNA expressing hybrid spike proteins along with SIV gag mRNA at a 1:2 ratio to generate VLP formation. Immune responses were more effective in the case of an mRNA vaccine co-expressing a truncated spike with SIV Gag [54]. The mechanisms underlying the increased immune responses are not clear. However, multiplex antigenic presentation, presentation of membrane-anchored proteins in their native conformation, and the presence of more than one antigenic protein could be possible reasons for increased immune responses by the hybrid vaccine platforms [54]. In another study, it was shown that truncation at the C-terminal site of the hepatitis B virus (HBV) core (HBc) protein is the deciding factor for RNA degradation. The HBc protein consists of a self-assembly domain (aa 1–140) and a protamine-like polyarginine (PA) domain (aa 150–183). HBc1-149 lacks all the C-terminal Arg blocks. In total, there were four arginine blocks in HBc (block1-HBc1-162, block 2-HBc1-163, block 3-HBc1-171, and block 4 - HBc1-175). The polyarginine domain is responsible for viral replication, the encapsidation of RNA, and the packaging of partially double-stranded genomic DNA. Elimination of the PA domain reduced RNA degradation. The presence of three arginine blocks led to 85% RNA degradation. However, the presence of all four arginine blocks slightly increased the encapsidation. The loss of encapsidation switches the Th2 immune responses to the Th1 type and decreases the levels of IFN-γ induction, which is a measure of the potential CTL activity of immunogens. The decrease in IFN-γ synthesis with C-truncation could be due to the loss of potential CTL epitopes [55]. These studies suggest that truncations at C t C-terminal site of proteins could be an attractive tool for further development of efficient vaccines [55]. A similar approach was used to form an mRNA vaccine against simian-human immunodeficiency virus infection. For example, a messenger RNA vaccine that co-expresses HIV-1 envelope (env) and SIV Gag proteins after transfection in female human embryonic kidney (HEK) 293T cells can generate virus-like particles (VLPs) that induce humoral immunity with broad neutralization and reduce the risk of simian human immunodeficiency virus infection in rhesus macaques [56]. These results suggest that Gag-based VLP-mRNA vaccines can be easily designed and are effective against several infectious diseases of global relevance. Moreover, the human genome contains genes that encode retroviral gag-like proteins. These proteins belong to the paraneoplastic Ma antigen (PNMA) family. PNMA2 is strongly secreted as an icosahedral capsid from human cells and can self-assemble from recombinant proteins, but lacks the capacity to package RNA. Recently, PNMA2 was engineered to incorporate nucleic acids and thus can be used as a delivery vehicle in mammalian cell lines, demonstrating the potential of engineered PNMA2 as a delivery tool for nucleic acids [57].

The biophysical attributes of VLPs, such as morphology, size, and polydispersity, determine their potency and safety of VLPs vaccines. It was recently shown that RNA can control the polydispersity of VLPs [58]. They explained this using a statistical model of viral self-assembly. According to this model, capsid formation in the absence of viral RNA is controlled by the balance between enthalpic (free energy gained by individual proteins to form each capsid) and entropic effects (number of ways to assemble these capsids at constant internal energy). Larger entropy favours small-sized homogenous particles because more small particles can be made than large particles at a constant number of total proteins [58]. Similarly, VLPs constructed from Gag proteins lacking residues 16–99 and the p6 domain and DNA assembled around spherical nanoparticles demonstrate reduced polydispersity, which suggests that size dispersion can be determined by the nucleic acid content of VLPs [59].

It was also shown that VLPs derived from the bacteriophage MS2 showed high stability and may serve as an improved platform for RNA-vaccine delivery. These VLPs have MS2 coat protein (MCP) containing nucleocapsid protein that recruits MS2 hairpin-containing mRNAs into the VLP. For instance, interaction of an RNA aptamer and the coat protein of bacteriophage MS2, successfully generate MS2 VLPs and addressed the questions of RNA stability, cost and limited production [60]. In these vaccines target mRNA is packaged by MS2 capsid protein. These vaccines have shown clinical value in the prevention and therapy of prostate cancer. A new RNA vaccine platform based on MS2 virus-like particles was developed in Saccharomyces cerevisiae [61]. Similar to MS2, other aptamers have also been used for VLP-mediated RNA or ribonucleoproteins (RNPs) delivery. For instance, aptamers PP7 and PP7 coat protein (PCP) in bacteriophage PP7, aptamer box-B and ABP λ peptide N22 in bacteriophage λ, and RNA aptamer com and its interacting protein Com in the bacteriophage Mu have been used for RNA imaging, DNA labelling, and dead cas9 mediated gene regulation [62]. The mRNA-VLPs have been produced for various RNAs, such as Cas9, Cre, cas9-sgRNA complex, and nano-luciferase (nLuc) mRNA [63–65]. VLPs containing sgRNA are called RNA-VLPs, whereas VLPs containing RNPs are called RNP-VLPs. These RNA-VLPs or RNPs-VLPs are used for the delivery of sgRNA, cas9-sgRNA complex, co-delivery of cas9-sgRNA and vector RNA, cas9-base editors RNPs, and co-delivery of cas9-mRNA and sgRNA. A common issue in VLP-mediated genome editing delivery vehicles is the requirement of viral envelope proteins to facilitate cell entry and endosomal escape. The most common viral envelope protein is VSV-G, which is toxic. In addition, any pre-existing immune response to VSV-G reduces the efficiency of gene editing. Therefore, the use of other non-toxic viral envelope proteins, such as the mouse SYNA protein, is required to increase the efficiency of gene editing. However, the use of non-viral proteins improves safety and efficiency [62].

In another approach of forming VLP-mRNA vaccines, that antisense RNA encoding foot-and-mouth virus (FMDV) genes are packaged into VLP. These VLPs present EP141–160 on its surface and protect from the foot-and-mouth disease. The results were encouraging in terms of protecting 40% of suckling mice and 85% (17/20) of guinea pigs from FMDV. However, current methods only allow the usage of VLPs to deliver linear mRNAs [66].

Delivery of circRNAs by VLPs can be more advantageous in comparison to linear mRNA. To the best of our knowledge, only one literature is available where VLPs are used to deliver circRNAs which were produced using the tornado system. Using this approach, they showed that the VLPs produced using this approach can enhance the durability and protein expression of circRNAs, thus demonstrating the usage of this technology for CircRNA delivery. To package circular mRNAs, they have used an envelope plasmid; a transfer plasmid and an integrase deficient packaging plasmid that expresses MS2 coat protein. The transfer plasmid was created by cloning an MS2 stem loop into the 3'UTR of the cytomegalovirus (CMV)-CVB3 Tornado translation system that expresses a nano-luciferase gene. CMV-CVB3 system contains Coxsackievirus B3 (CVB3) IRES. Due to the presence of MS2 sequence in transfer plasmid, RNA is packaged into VLPs by interacting with the MS2 coat protein. They showed that VLPs produced using the Tornado translation system enhance protein expression compared to conventional VLPs [8]. However, in this system in-vitro transcription is required using mammalian cells which could be the limitations in large scale production of vaccines at low cost.

## VLP-CircRNA as a dual vaccine candidate

VLPs are used as a chimeric vaccine candidate where it can express multiple antigens on its surface. Thus, a single chimeric VLP can induce immune response to several infectious diseases. Chimeric VLPs are generated by fusion technology that offer broad protection against different viral strains. For example, receptor binding domain (RBD) domain of SARS-CoV-2 was fused to the C terminus of AP205. AP205 is a VLP that displays the RBM on its surface. It was shown that immunization of mice with fused VLP generates high levels of IgG antibodies recognizing both RBD and spike protein of SARS-CoV-2 [67]. Similarly, a VLP-based COVID-19 vaccine candidate, PRAK-03202 was generated where co-expression of the spike, envelope, and membrane proteins was done using a highly characterized *S. cerevisiae*-based D-Crypt™ platform. This system allows scalability of the drug product, and thus circumvents conventional vaccine production complexities in eggs or cell culture [68]. The D-crypt™ platform has a low risk of contamination by adventitious agents, low production costs, and the ability to produce VLPs of reliable quality and quantity. VLPs have been successfully used as a vaccine platform to which multiple proteins can be attached or inserted and shown to stimulate both cellular and humoral immunity [69]. These vaccines have several advantages, such as safe production process, short production time, and many available expression systems. VLP-based vaccines are commercially available against various pathogens, such as zika virus, hepatitis C virus (HCV), hepatitis B virus (HBV), and human papilloma virus (HPV) [70].

Thus, it would be interesting to explore the usage of innovative platforms generating VLP-circRNA vaccine as a vaccine candidate along with the delivery vehicle to target multiple infectious diseases or a single infectious disease caused by the different strains of microorganisms at a glance. The usage of VLPs in circRNA vaccines will also improve the bioavailability of circRNAs. However, VLPs based vaccines showed many problems such as purification, and scale-up production to be solved in the development of effective vaccines and carriers. Therefore, development of new platforms for generating scale-up production of CircRNA-VLP vaccines are needed.

## Platform for the synthesis of CircRNA-vlp dual vaccine

**Figure 4.**
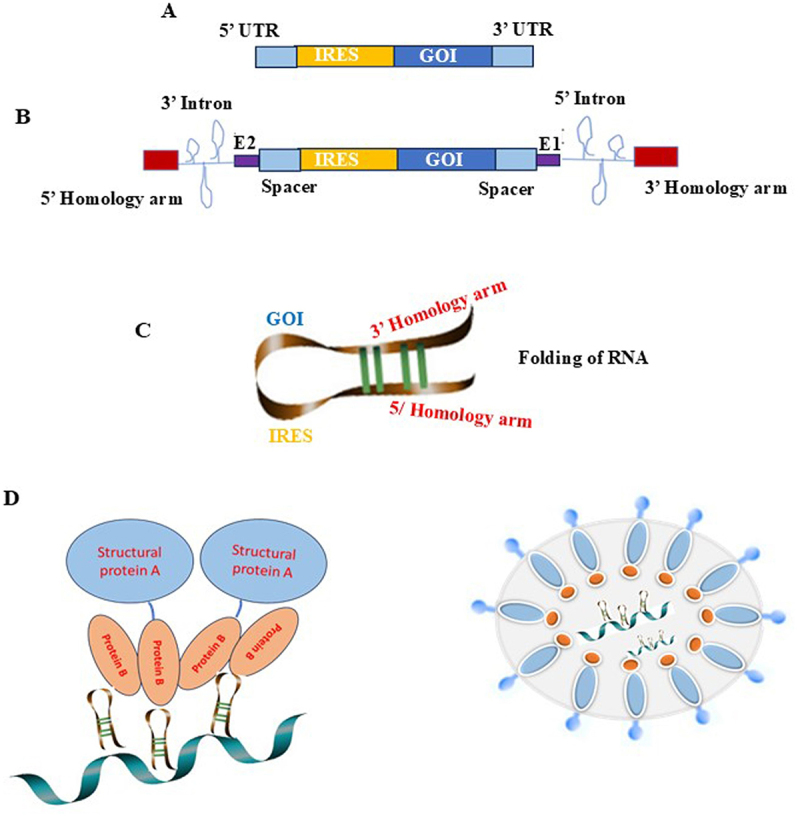
Messenger RNA encapsulation in VLPs. a) sequence of linear mRNA b) sequence of circRNAs containing self-splicing introns and homology sequences c) circularized RNA transcript showing stem loops for the interaction with coat protein d) interaction of circRNA with coat protein of VLP and encapsulation of circRNA in VLP.

To the best of our knowledge only one platform, D-crypt, has been shown to produce VLP-circRNA dual vaccine against Respiratory Syncytial Virus (RSV) and influenza. However, the data is unpublished. D-crypt platform developed by Premas Biotech has been shown to generate RSV CircRNA encapsulated in Flu VLP in the space of respiratory diseases https://www.premasbiotech.com/vaccines.html. The D-Crypt platform combines a protease-deficient strain of Bakers' Yeast, *Saccharomyces cerevisiae* expression host with a collection of more than 20 custom made expression vectors [68,71]. The vaccine candidate comprises three antigens (HA, NA, and M1) of H1N1 as the VLP proteins and the key fusion (F) glycoprotein (PreF) of RSV present as the encapsulated CircRNAs. PreF-CircRNA, transcription, expression, and encapsulation design incorporate internal ribosome entry sites (IRES) and open reading frame (ORF) for the expression of cloned PreF, along with sequence elements leading to its circularization through self-spliced intron and back splicing (Figure 4). The resultant CircRNA-VLP vaccine candidate was shown to have increased stability by means of protection from degradation by RNases and imparts thermal stability (data not published). It was also shown that the D-crypt platform is capable to scale up production of vaccines for further studies [71].

## Future considerations and conclusion

In summary, it is indispensable to develop novel methods for increasing the circularization efficiency of circRNA vaccine, synthesis, purification, and delivery. These improvements will aid in the process of treatment and prevention of diseases using circRNA-vaccines. Notably, the role of VLPs as a delivery vehicle is at the preliminary stage for circRNAs. It should be further explored in terms of increasing circularization efficiency of CircRNAs, avoidance of IVT and encapsulation or exploration of platforms increasing the scale-up production of CircRNA vaccines. We propose that new platforms should be developed that can overcome the drawbacks of current platforms, for example, avoidance of chemical modification, identification of novel discovery tool etc (Table 2). We also believe that VLPs in CircRNA-VLPs should also be explored as a vaccine candidate to generate dual vaccines to target multiple diseases where circRNA packaged in VLP can target one disease and VLP can target another disease.

**Table 2.** Comparison of m-RNA vaccine platform.

The competitive landscape RNA Vaccines		
Parameters	RNA vaccines (Current platforms)	RNA Vaccines (Proposed platforms)
Methods	In-vitro transcription (IVT) is required for the synthesis of mRNA or circRNA. Chemical modification of circRNA such as m6A modification at 5’UTR regions in in vitro methods	In vivo technology ‘In cell’ RNA transcription and encapsulation No chemical modification required Circularization occurs via self-splicing introns and homology arms.
Production	Mammalian expression system Multistep to produce final drug product Difficulty in manufacturing, High production costs Sensitivity to temperature and stability rendering it inaccessibility to LIMC countries.	Low-cost expression system Fewer steps Easy manufacturing Scalable with high yields Low COGs Thermostable hence aids in solving transportation issues
Delivery and Safety	In most of the RNA vaccines delivery is done by LNPs, which show high reactogenicity. mRNA based vaccines are found to comprise most serious side-effects, albeit occurring in sparing numbers	Suitable delivery vehicle such as VLP having no reactogenicity and safe
Formulation	Adjuvants may or may not be required	Not required and that may help in reducing the adjuvant-based toxic effects.
